# Impact of a strategy based on unique blood culture sampling on contamination rate and detection of bloodstream infections in critically ill patients

**DOI:** 10.1186/s13613-023-01107-y

**Published:** 2023-03-03

**Authors:** Rafael Mahieu, Carole Lemarié, Delphine Douillet, Alain Mercat, Hélène Cormier, Matthieu Eveillard, Vincent Dubée, Jérémie Riou, Achille Kouatchet

**Affiliations:** 1 Department of Infectious Diseases, Angers University Hospital, 4 Rue Larrey, 49933 Cedex Angers, France; 2 CRCINA, Inserm, Univ Angers, Université́ de Nantes, SFR ICAT, 49000 Angers, France; 3 Laboratoire de Bactériologie, Centre Hospitalier Universitaire d’Angers, Angers, France; 4 Emergency Department, Angers University Hospital, Univ Angers, Angers, France; 5 UMR MitoVasc CNRS 6015 - INSERM 1083, Health Faculty, Univ Angers, FCRIN, INNOVTE, Angers, France; 6 Department of Medical Intensive Care, University Hospital, Angers, France; 7 MINT, UMR INSERM 1066, UMR CNRS 6021, UNIV Angers, Micro Et Nano Médecines Translationnelles, Angers, France; 8 Methodology and Biostatistics Department, Delegation to Clinical Research and Innovation, Angers University Hospital, 49100 Angers, France

**Keywords:** Unique blood culture, Blood culture, Sampling procedure, ICU, Bacteremia

## Abstract

**Background:**

Unique blood culture (UBC) has been proposed to limit the number of venipuncture and to decrease the risk of BC contaminations (BCC) without affecting their yield. We hypothesized that a multi-faceted program based on UBC in the ICU may reduce the rate of contaminants with a similar performance for bloodstream infections (BSI) identification.

**Methods:**

In a before and after design, we compared the proportion of BSI and BCC. A first 3-year period with multi-sampling (MS) strategy followed by a 4-month washout period, where staff received education and training for using UBC, and a 32-month period, where UBC was routinely used, while education and feedback were maintained. During the UBC period, a large volume of blood (40 mL) was sampled through a unique venipuncture with additional BC collections discouraged for 48 h.

**Results:**

Of the 4,491 patients included (35% female patients, mean age 62 years) 17,466 BC were collected. The mean volume of blood per bottle collected increased from 2.8 ± 1.8 mL to 8.2 ± 3.9 mL between the MS and UBC periods, *P* < 0.01. A 59.6% reduction (95% CI 56.7–62.3; *P* < 0.001) of BC bottles collected per week was observed between the MS and UBC periods. The rate of BCC per patient decreased between the two periods from 11.2% to 3.8% (73.4% reduction; *P* < 0.001) for the MS and UBC periods, *P* < 0.001. Meanwhile, the rate of BSI per patient remained stable at 13.2% and 13.2% for the MS and UBC periods, *P* = 0.98.

**Conclusions:**

In ICU patients, a strategy based on UBC reduces the contamination rate of cultures without affecting their yield.

**Graphical Abstract:**

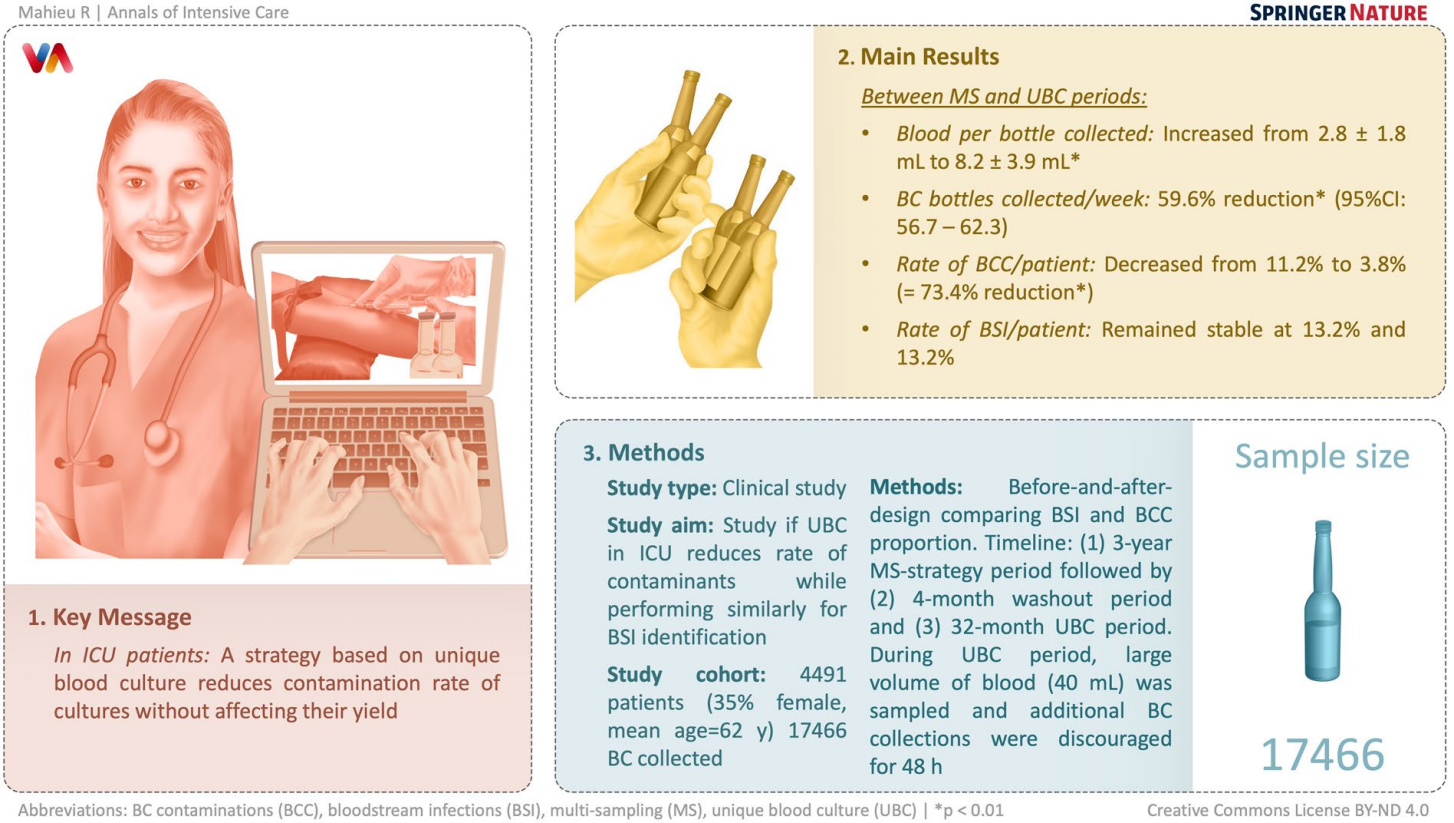

**Supplementary Information:**

The online version contains supplementary material available at 10.1186/s13613-023-01107-y.

## Introduction

A bloodculture (BC) collection usually includes one aerobic bottle, and one anaerobic bottle; this pair of bottles is conventionally referred to as a BC set. About 1 to 2.4% of these sets of BC are false positives (BC contaminants, (BCC)) which represent 12 to 34% of positive BC [1–3]. To optimize the rate of bloodstream infections (BSI) identification while maintaining a low rate of BCC, several measures have been recommended. First, inappropriate ordering of BC collection is frequent and sampling protocols based on the sole criterion of fever are associated with a high rate of BC. These inappropriate BC samplings are thus associated with unnecessary antibiotic prescriptions, additional workload for phlebotomists, additional discomfort for the patient and, overall, additional costs [4, 5].

For decades, strategies to improve the performance of BC (detection of true bacteremia and limitation of BCC) have focused on hygiene’s procedures and laboratory techniques with the development of new BC systems and culture media [6]. However, optimizing the way BC are collected may be an inexpensive and efficient strategy. Given the possibility of intermittent bacteremia, the standard approach is the multi-sampling (MS) strategy, which implies obtaining BC sets from two to three separate venipunctures within a short period [7]. However, there is limited evidence in the existing literature to support the concept of intermittent bacteremia, whereas the volume of blood collected appears to be the most important parameter to identify a BSI [8–12]. Moreover, real-life use of MS procedures often result in a low number of BC collection. A single BC set (i.e., culture of only 2 bottles also named solitary BC) is obtained in up to 41% to 94% of patients when MS procedure is used [1, 13, 14]. Another concern with the MS procedure is the rapid and significant loss of pathogen detection after initiation of antibiotic therapy [15]. For patients requiring rapid initiation of treatment, subsequent BC are frequently sampled after administration of the first antibiotic dose, which decreases their diagnostic yield.

A unique BC (UBC) strategy, corresponding to a large amount of blood drawn during a unique venipuncture, may combine the benefits of an optimal blood volume collection with a decreased risk of BCC. In the emergency department (ED), a UBC strategy has demonstrated a similar performance for the identification of BSI with a lower rate of BCC when compared to MS strategy [1]. However, a UBC strategy has not been evaluated in the intensive care unit (ICU) with the specific problem of a high incidence of fever during the ICU stay. Predicting a BSI in a febrile and critically ill patient (at admission or during ICU stay) is difficult, with only 10% of patients sampled having a BSI [1, 2, 16]. Changing a MS procedure for a UBC strategy without focusing on the ordering rule for BC collection would be associated with an important increase in the total number of collected BC bottles.

In this study, we hypothesized that a multi-faceted program based on a UBC strategy in the ICU, at admission and all along ICU stay, may optimize the detection of BSI with a reduction of BCC; we thus compared the rate of BSI and BCC during a 6-year period in an ICU, before and after implementing such program.

## Methods

### Setting and population studied

The study was conducted in a 24-bed medical ICU from January 1st, 2013 to December 31th, 2015 (MS period) and from April 1st, 2016 to December 31th, 2018 (UBC period). All critically ill adult patients with a least one BC collection and hospitalized in the ICU during the study period were included. Data on patients, blood cultures (number of sets, number of bottles, rate of pathogens and BCC) and volume sampled were prospectively monitored.

### Study design

#### Multisampling period

A set of BC (1 aerobic + 1 anaerobic bottle) was collected by ICU nurses in case of fever above 38.5 ℃ (101.3°F), and/or chills, and/or on medical order because of patient status worsening. Additional BC are drawn without medical order when fever occurs (above 38.5 ℃) with a maximum of 3 BC sets/day. This procedure is also implemented in the emergency department and in most medical wards. No medical order is needed to collect these BC for most patients.

#### Unique blood culture period

The implementation of the UBC strategy was a multi-faceted educational program including a standardized training program, the UBC protocol and a nurse survey on the knowledge on BC (Protocol Additional file 1: Table S1, Table S2 and Table S3). Medical staff (physicians and residents) and ICU nurses were trained during a 1-h standardized training program provided by the same intensivist (M.R.) during the whole study. The UBC protocol was posted in the admission area and in the ICU subunits with a reminder of the importance of the blood volume collected (up to 10 mL per bottle) and hygiene procedure.

The UBC consisted of sampling a large volume of blood (40 mL) through a unique venipuncture and equally distributed into 2 aerobic bottles and 2 anaerobic bottles (each bottle filled with 10 mL of blood). BC collection could only be performed on medical prescription. Collecting and/or prescribing additional BC was strongly discouraged for at least 48 h. This point was emphasized during the training of prescribers and nurses and was considered as a major change in the way BC were collected.

The educational program was repeated every 6 months (shift of new residents) and administered to every new paramedic or physician in the ICU (Additional file 1: Figure S1).

### BC definitions and indicators

A BC collection was defined as a collection of blood during a unique sample procedure (one venipuncture) whatever the number of BC bottles collected. A solitary BC was defined as a collection of only 2 bottles during a 24-h period [1, 13, 14]. The rate and incidence of BSI and BCC for 1000 patient-days were compared between the two periods.

### Definition of BC contaminants

A BCC was defined as a BC with only one bottle growing skin organisms (coagulase-negative staphylococci (CoNS), viridans group streptococci, *Corynebacterium* spp., *Cutibacterium* spp. (ex. *Propionibacterium* spp.), *Bacillus* spp. or *Micrococcus* spp) with no clinical suspicion of a specific portal of entry and for which no definitive antimicrobial therapy was prescribed [4, 17]. For suspicion of central line-associated BSI including a skin organism, medical records were independently reviewed by two physicians (RM and AK). Disagreements were resolved by a third physician reviewer.

### BC device and estimation of blood volume collected

The volume of blood sampled during BC collection was assessed during seven pre-specified 1-month periods. During the same periods, the blood volume sampled during BC collection was also evaluated in the ED, to assess trends unrelated to the study at the hospital level. The volume was determined for each filled bottle using the following formula (considering the density of blood was 1055 g/ml) [18].

$$Blood\,volume\,\left(ml\right)=\frac{weight\,of\,filled\,bottle\,\left(mg\right)-average\,weight\,of\,empty\,bottles\,(mg) }{blood\,density\,(mg/ml)}$$

BC bottles were incubated using the automated BACT/ALERT® VIRTUO® system (BactAlert FA/FN plus, bioMérieux, Marcy l’Etoile, France).

### Statistical analysis

All statistical analyses were performed using R Core Team software (4.0.3).

For descriptive analysis, quantitative variables were reported as mean ± standard deviation (SD) when their distribution can be considered as Gaussian, and with median and inter quartile ranges (IQR) otherwise. Qualitative variables were reported using effective (n) and percentage (%).

Fisher’s exact test were used to study the association of categorical variables and period. *T* test were used to study the association of gaussian continuous variables and period, otherwise when the distribution cannot be considered as Gaussian, Mann–Whitney test was performed. The proportion of BSI and BCC and their 95% CI were compared between the MS period and the UBC period, CI95 was also provided. All tests were two-sided.

A *P* value below 0.05 was considered statistically significant.

To explain the evolution of binary variables over time as a function of the period, mixed effects logistic regression models with fixed effect on period (MS vs UBC) with a random effect on time (weeks) were performed.

To explain the evolution of counting variables over time as a function of the period, generalized Poisson regression models with fixed effect on period (MS vs UBC) with a random effect on time (weeks) were performed.

Loess regression is a non-parametric approach using local weighted regression to fit a smooth curve through the points of a scatterplot. Loess curves can reveal trends in the data that might be difficult to fit with a parametric curve. Then, to study the trends in the incidence of bloodculture, bacteremia and contaminants for each study period were summarized using this approach.

No imputation of missing data was performed. Holm procedure was used to control the Family Wise Error Rate in the context of multiple testing."

### Ethical considerations

The study obtained approval from Angers University Hospital ethical committee (N°2016/65), and from the French Commission Nationale de l'Informatique et des Libertés (N° 2018-043). The need for an individual informed consent was waived.

## Results

### Study population

**Fig. 1:**
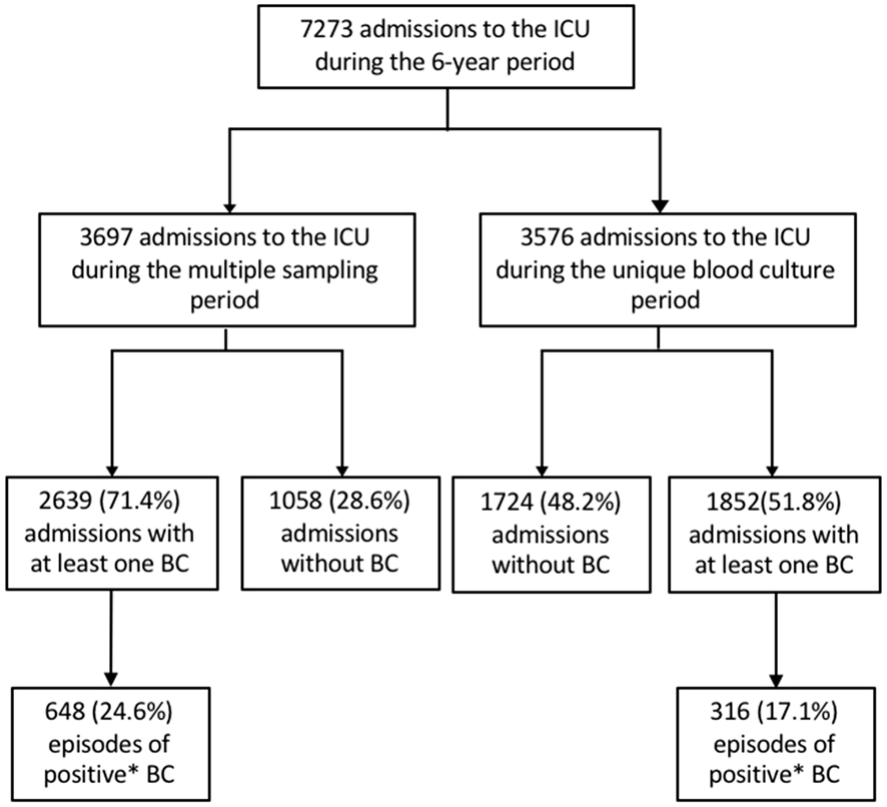
Enrolment of patients. *A positive BC refers to a bloodstream infection or a BC contaminant

During the study period, 7,273 ICU patients were hospitalized (Fig. 1). At least one BC was collected in 61.7% of patients (*n* = 4,491), corresponding to 17,466 BC collections and 35,460 patient-days. The characteristics of the patients are presented in Table 1. During the UBC period, patients were older (63 ± 16 vs 62 ± 17 years), had a higher SOFA score (7 ± 4.3 vs 6.6 ± 4) and SAPS II score (48 ± 21 vs 44 ± 19), had higher rate of medical reason for admission (98% vs 94%), longer duration of mechanical ventilation (6 ± 11 vs 5 ± 10) and higher rate of central line catheter (57% vs 52%).

**Table 1:** Characteristics of the patients

Characteristics	Overall (*n* = 4491)	MS period (*n* = 2639)	UBC period (*n* = 1852)	*P* value
Age—yr	62 ± 17	62 ± 17	63 ± 16	0.02
Male sex, n (%)	2901 (65)	1678 (64)	1223 (66)	0.1
Simplified Acute Physiology Score II	45 ± 20	44 ± 19	48 ± 21	< 0.001
Sequential Organ Failure Assessment score	6.7 ± 4.1	6.6 ± 4	7 ± 4.3	0.001
McCabe score, *n* (%)				< .001
1	2029 (45)	1348 (51)	681 (37)	
2	1727 (38)	841 (32)	886 (48)	
3	735 (16)	450 (17)	285 (15)	
Reason for admission, *n* (%)				<0 .001
Medical	4276 (95)	2469 (94)	1807 (98)	
Scheduled surgery	25 (0.6)	20 (0.8)	5 (0.3)	
Emergency surgery	190 (4.2)	150 (5.7)	40 (2.2)	
Mechanical ventilation, *n* (%)	2880 (64)	1716 (65)	1164 (63)	0.14
Duration—days	6 ± 10	5 ± 10	6 ± 11	0.007
Central venous catheter, *n* (%)	2426 (54)	1364 (52)	1062 (57)	<0 .001
Duration—days	8 ± 18	7 ± 17	9 ± 19	< 0.001
Indwelling urinary catheter, *n* (%)	3799 (85)	2222 (84)	1577 (85)	0.4
Outcomes				0.3
In-ICU death	1056 (24)	616 (23)	440 (24)	0.8

### BC collection

**Fig. 2:**
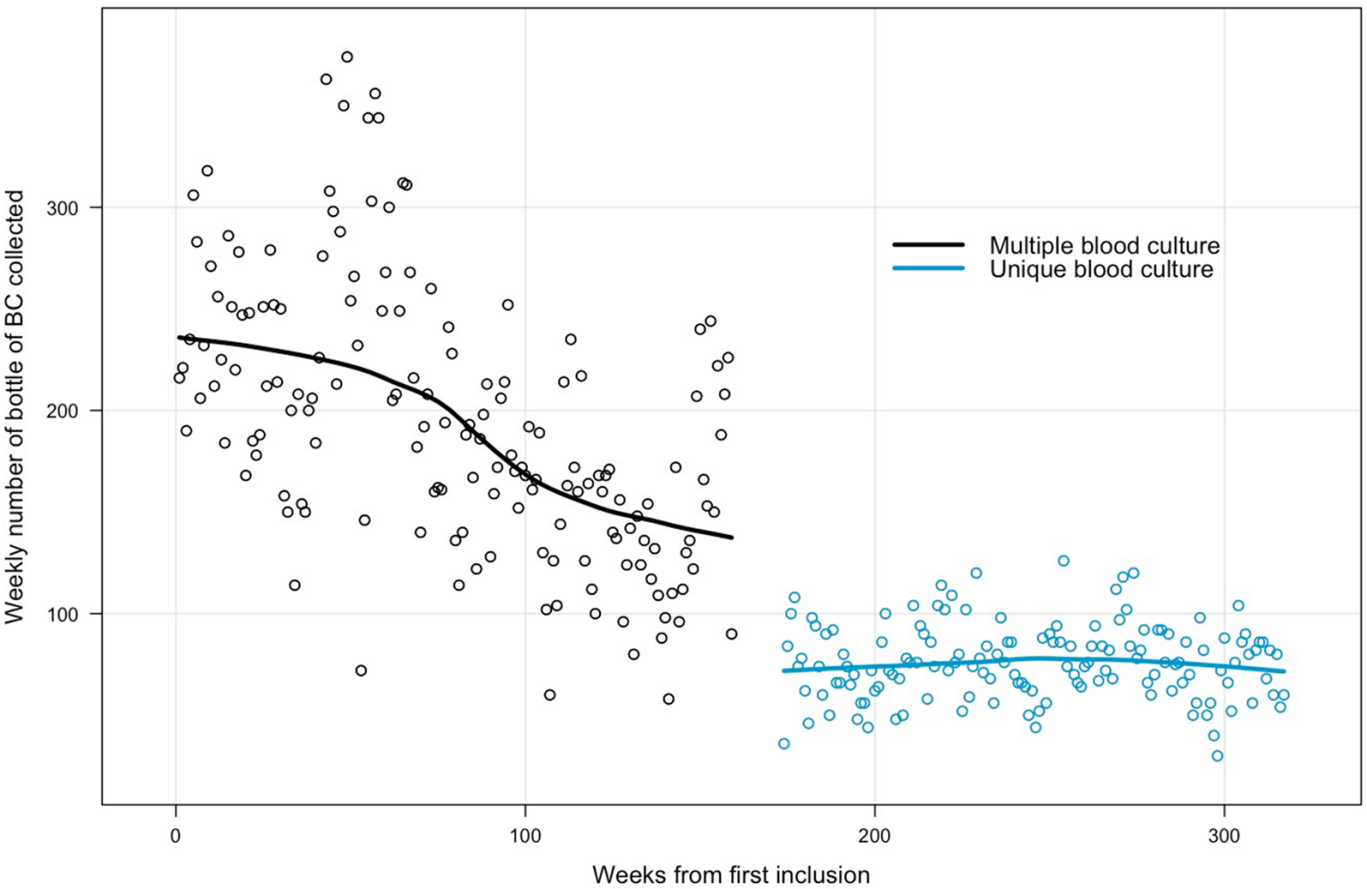
Weekly variations of BC bottles collected. Mean number of bottles of BC collected per week was significantly lower during the unique blood culture period than during the multi-sampling period (76 (95% CI 73–79) and 196 (95% CI 186–206), *P* < .001)

A 59.6% reduction (95% CI 56.7–62.3, *P* < 0.001) of BC bottles collected per week was observed (Fig. 2). The number of venipunctures for BC per 1,000 patient-days decreased by 78% (Table 2). The details of BC results are presented in Table 2

**Table 2:** Number, rate and incidence of bloodstream infections and contaminants for each period

Characteristics	Overall	MS period	UBC period	*P* value
ICU patients	7273	3697	3576	
ICU patients with BC collection	4491	2639	1852	
Cumulative ICU days	35,460	19,292	16,177	
BC sets	17,466	14,735	2731	
Bottles of BC	41,251	30,444	10,807	
BC sets/patients	3.9 ± 7.2	5.6 ± 9	1.5 ± 1.1	<0 .001
BC sets per 1000 patient-days	411	764	169	< 0.001
Bottles of BC/patients	9 ± 15	12 ± 19	6 ± 5	< 0.001
Bottles of BC per 1000 patient-days	971	1578	668	<0 .001
Positive BC (pathogens and BCC)	959	643	316	<0 .001
BCC				
Number	366	295	71	–
Rate by BC sets, % (95% CI)	2.1 (1.9–2.3)	2.0 (1.8–2.2)	2.6 (2.1–3.2)	0.06
Rate by patient	8.1 (7.4–9)	11.2 (10–12.5)	3.8 (3–4.8)	<0 .001
Per 1000 patient-days	10.3 (9.3–11.3)	15.3 (13.6–17.1)	4.4 (3.4–5.5)	< 0.001
Rate among positive BC	38.2 (35.2–41.4)	45.9 (42.1–50)	22.5 (18.1–27.6)	< 0.001
Rate by bottle of BC	1.1	1.1	0.9	0.1
BSI				
Number	593	348	245	–
Rate by BC sets, % (95% CI)	3.4 (3.1–3.7)	2.4 (2.1–2.6)	9 (7.9–10.1)	<0 .001
Rate by ICU stay	13.2 (12.2–14.2)	13.2 (11.9–14.6)	13.2 (11.7–14.9)	> 0.99
Per 1000 patient-days	16.7 (15.5–18)	18 (16.1–20.1)	15.1 (13.3–17.1)	0.1
Rate among positive BC	61.8 (58.7–64.9)	54.1 (50.2–58)	77.5 (72.2–81.7)	<0 .001
Rate by bottle of BC	4.3	3.6	2.2	< 0.001

### BSI and BCC

**Fig. 3:**
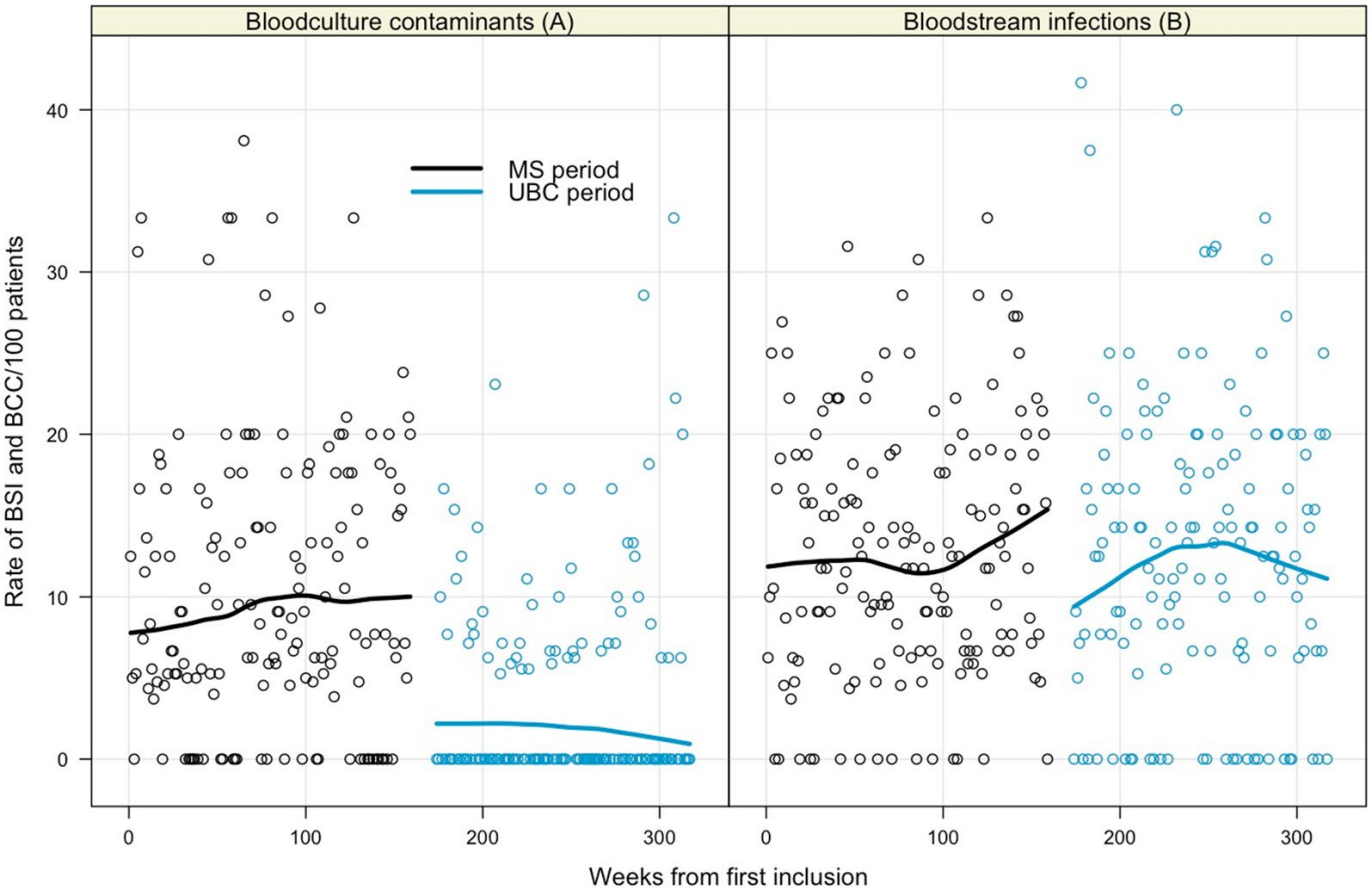
Rate of BCC and BSI per week between the MS and UBC periods. 3**A**: the rate of BCC per week decreased by 62% from (10.4% to 3.9%) between the MS and UBC periods (*P* < .001). 3**B**: the rate of BSI per week was similar between the MS and UBC periods (*P* = .91). MS: multi-sampling; UBC: unique blood culture

Overall, a BSI was highly suspected in 21.4% of patients (959 episodes of positive BC for 4,491 patients with at least one BC). The rate of BCC and BSI over time are presented in Fig. 3. The rate of BC contaminants decreased from 11.2 to 3.8 per 100 patients (reduction of 73.4%, 95% CI 58.1–88.8, *P* < 0.001) between the MS and UBC periods. Meanwhile, the rate of BSI remained stable at 13.2% and 13.2% during the MS and UBC periods, *P* = 0.98.

The yearly variation of BSI and BCC rate are presented in Additional file 2: Figure S2.

Among positive BC, the proportion of pathogens increased from 54.1% (95% CI 50.2–58) during the MS period to 77.5% (95% CI 72.2–81.7) during the UBC period (absolute difference of + 23.4% [95% CI 17.1–29.6], *P* < 0.001). This proportion of pathogen and contaminant among positive BC over time are represented in Additional file 2: Figure S3.

*Enterobacteriaceae*, *Staphylococcus aureus* and *Streptococcus* spp*.* were the most frequent bacteria identified with no difference between the two periods, *P* = 1 (Additional file 2: Table S4). No significant difference was observed for the ratio of community-acquired and nosocomial infections (*P* = 0.3) and for the rate of BSI due to Gram-negative (42.2% and 41.4%, for the UBC and MS periods, *P* = 0.8) or Gram positive (48.8% and 50.3%, for the UBC and the MS periods, *P* = 0.7) between the two periods.

Among the 1,056 in-ICU deaths, no difference was observed for the rate of BSI (18.4 and 14.9 per 100 patients for the UBC and the MS periods, *P* = 0.13). Meanwhile, the rate of BCC for these patients decreased from 9.4 to 4.3 per 100 patients (reduction of 54.4%, *P* = 0.003) between the MS and UBC periods.

Of the deceased, an infection was the reason for ICU admission in 39 and 27%, *P* < 0.001 for the MS and UBC periods, respectively.

### Blood volume collected and correlation with BSI

Four thousand bottles of BC (9.7% of the 41,251 bottles) were manually weighted. During the MS period, the mean volume of blood collected per bottle was 2.8 ± 1.8 mL in the ICU, and 2.3 ± 2.7 mL in the ED, *P* = 0.11. After the implementation of the UBC strategy in the ICU, the mean volume of blood increased to 8.2 ± 3.9 mL (*P* < 0.01 for comparison with MS periods), whereas it remained stable in the ED (Additional file 2: Figure S4). No significant difference in the volume of blood collected per bottle was observed between the early (M + 2) and late (M+24) period of the UBC strategy in the ICU (*P* = 0.3).

A strong positive correlation was observed between the volume of blood collected and the identification of a BSI in the ICU (Additional file 2: Figure S5). For each additional milliliter of blood collected in a bottle of BC, the rate of positivity increased by 8% (95% CI 5–11), *P* < 0.001.

### Nurse survey: knowledge on blood culture

Seventy eight percent of ICU nurses (68/87) were evaluated on the two baseline surveys and 79% of these (54/68) 6 months after the educational program. Overall, the rate of correct answers increased from 68 to 83% between baselines and 6-month surveys, *P* < 0.001 (Additional file 2: Figure S6).

## Discussion

In this 6-year before–after study in patients hospitalized in ICU, a multi-faceted program based on a unique sampling of a large volume of blood for diagnosing BSI (UBC) instead of a conventional multiple sampling (MS) procedure was associated with a major decrease in the number of venipunctures per patient, with a similar rate of BSI identified, while the rate of contaminated blood cultures per patient decreased from 11.2 to 3.8%. The increase in the volume of blood collected and a lower number of venipunctures per patient might explain the performance of the UBC strategy.

### Sampling strategy and pathogen identifications

Seminal studies in the 1970s reported that serial blood cultures taken within 24–48 h were able to confirm 99% of BSI [19, 20]. Li et al. (1994) observed that, for a similar volume of blood collected, no advantage was observed with any particular interval of collection [11]. The concept that most cases of bacteremia were continuous rather than intermittent emerged, and the first study of a unique venipuncture for detecting BSI was published in 1996, showing a good identification rate of pathogens [21, 22]. More recently, the UBC strategy (40 mL of blood equally distributed into two aerobic bottles and two anaerobic bottles) was compared to a MS strategy in the ED with a 97.4% and 95.5% rate of pathogen detection (each patient being his own control) [1]. No studies on UBC have been conducted to date in intensive care wards. In our study, the incidence of BSI was identical between the two periods at 13.2%, close to the 8.2–11% BSI rate found in the literature in the ED or for ICU admission [1, 23–25].

Notably, the BSI rate did not decrease despite a major reduction of BC sets obtained. A similar result was found by Dargère et al. [1], showing that the UBC strategy had a net clinical benefit illustrated by a higher sum of BSI missed and BCC with the MS strategy).

### Volume

Most BSI in adults have a low bacterial inoculum and the volume of blood collected for culture appears to be the most important parameter to identify bacteremia [21, 26]. Overall, about one half of patients with a BSI have a bacterial concentration below one CFU/mL [26]. For example, a bacterial density below one CFU/mL has been described in 38% and 65% of patients with *S. aureus* and *E. coli* BSI [21]. The sensitivity of BC collection increases with the number of BC collected with 70–90% of BSI identified with the first BC set and an additional yield of 5–20% and 2.4–15.3% for the second and third BC set [1, 8, 10–12, 19, 27]. Because most studies did not monitor the volume of blood collected, the number of BC sets may not represent adequately the volume of blood cultured. In our study, the median volume of blood collected before implementing the UBC strategy was very low, with a median volume of 2.8 mL in the ICU and 2.3 mL in medical wards. Despite the apparent low quality of these BC collections, this result is consistent with a large study conducted in 10 U.S hospitals, with an average volume of 2.3 mL per bottle and no hospital collecting an optimal volume of blood [28]. The lack of previous educational program and the workload associated with an optimal filling of BC (estimated to 7 min per sampling for an adequate volume collected) probably explain these results [1]. An adequate volume of 40–60 mL (i.e., two to three set of BC when bottles are correctly filled up) has been associated with an optimal sensitivity and may be recommended [1, 9]. During the UBC period, we observed an increase in the blood volume collected in the ICU department (8.2 mL in the ICU vs 2.5 mL during the same period in medical wards) corresponding to a threefold increase in the volume of blood collected per bottle. This increase in the volume of blood collected per bottle might have compensated the decrease of the number of bottles collected. Indeed, we confirmed the increasing yield of bacteremia with increasing volume sampled for culture (Additional file 2: Figure S5).

### BC contaminants

The BCC rate markedly decreased from 11.2% to 3.8% per patient during the MS and UBC periods (*P* < 0.001). The consequence of an increased number of unnecessary BC in the ICU is well-illustrated by the study of Verboom et al. who implemented a routine BC collection in all patients (regardless of infection suspicion) in attempt to improve the rate of early detection of BSI [24]. They estimated that drawing blood from 17 patients was needed to identify one additional patient with BSI. However, a 4.2-fold increase in BCC was observed after their protocol implementation [24], from 2.3% of patients when BC was ordered only for clinically suspected infection to 9.6% during the routine BC collection period. This higher rate reflects the increased risk of contamination with each additional BC collection and venipuncture. Conversely, if additional BC bottles are sampled during the same venipuncture (such as in the UBC procedure), the BCC rate per patient decreases for a similar or higher amount of blood cultured. In a quite logical way in this study, the rate of BCC by BC set was not different between the two periods. This result probably reflects that the number of venipunctures is the parameter that predict the most the contamination risk.

### Effect of the educational program

For a new procedure, a multifaced approach has been associated with a higher rate of successful implementation [29]. Compared to the MS period, the rate of BSI and BCC in the UBC period may have been impacted differently by each of the measure included in this multifaced program. The weight of each intervention has not been evaluated and some of them may have strongly impacted the rate of BCC. However, we think that a comparison of each measure one by one would have been out of touch with reality.

### Strengths and limitations of the study

The 6-year period of the study allowed studying a large number of critically ill patients with a large number of suspected BSI. No previous study has been conducted in the ICU to compare different sampling procedures for BC collection. The prolonged period of active surveillance and the number of episodes of suspected BSI analyzed may provide a sufficient body of evidence to support a UBC strategy in the ICU.

The level of adherence in our study was high as shown by the high volume of blood sampled in each bottle (with sustained performance at the M + 24 evaluation) and the low rate of solitary BC collections. The compliance of health care workers to the new procedure usually require a multifaceted intervention program with repeated performance assessments and feedback [29]. Indeed, the implementation of our procedure was time consuming, and labor intensive. Whether this organization and feedback program can be conducted in other ICUs remains to be determined.

The before/after study design may be responsible of some limitations in this study. Some differences in patient’s characteristics were noted between the two study periods, such as for age and severity scores. However, these differences were numerically small (Table 1) and tended to favor the MS group. A significant difference was observed for the deceased in the rate of admission for infection reason without a particular explanation. A potential overall improvement in BC sampling procedures over time may also explain a lower rate of BCC. However, we controlled for the volume of blood collected for BC in the medical wards and noted no change during the 3-year period of UBC in the ICU. Similarly, there was no difference in the volume of blood collected in the ICU between the three baseline assessments conducted before the implementation of the UBC strategy.

Finally, like all study on BC, a bias in the classification of the patient having BSI or not is possible, because the BC is its own gold standard and some true bacteremia may have been missed in both periods.

## Conclusion

A strategy based on UBC appears to be a safe and effective strategy for BC sampling in critically ill patients. Provided less venipunctures are performed but a large amount of blood is sampled for each BC set to optimize the yield of BC, it markedly limits the risk of BC contamination and associated unnecessary consequences.

## Supplementary Information

**Additional file 1: Table S1.** Educational program content. **Table S2.** Comparison between Multi-sampling and Unique blood culture protocols. **Table S3. **Knowledge survey about blood cultures, bloodstream infections and contaminants (multiple choice questions). **Figure S1.** Schema of our multifaceted educational program for a unique blood culture strategy.

**Additional file 2: Figure S2. **Evolution of BSI and BC contamination rate during the MS and UBC periods. **Figure S3.** Proportion of pathogen and contaminant among positive BC. **Table S4.** Site of acquisition, source of infection and pathogen identifications of BSI. **Figure S4.** Comparative changes in the mean volume of blood collected per bottle during the MS and UBC periods in the ICU and in the emergency department (ED). **Figure S5.** Proportion of bacteremia identified according to different volume of blood collected. **Figure S6.** Mean rate of correct answer for each question of the survey about bloodculture.

## Data Availability

The data sets generated during and/or analyzed during the current study are available from the corresponding author on reasonable request.
