# Supplementary material for: Impact of a strategy based on unique blood culture sampling on contamination rate and detection of bloodstream infections in critically ill patients
Source: Ann Intensive Care. 2023 Mar 3;13:13. doi: 10.1186/s13613-023-01107-y (PMC9984630; doi:10.1186/s13613-023-01107-y)
Supplement: Supplementary file 1 — Additional file 1: Table S1. Educational program content. Table S2. Comparison between Multi-sampling and Unique blood culture protocols. Table S3. Knowledge survey about blood cultures, bloodstream infections and contaminants (multiple choice questions). Figure S1. Schema of our multifaceted educational program for a unique blood culture strategy. [file 13613_2023_1107_MOESM1_ESM.docx]

| **Table S1: Educational program content** | |
| --- | --- |
| **General information** | |
| Blood culture | BC is the gold standard to diagnose BSI but false positives (skin contaminants) are frequent |
| Volume | The volume of blood collected is the most important parameter to identify a BSI. Forty milliliters of blood are necessary to get the best diagnostic sensitivity with a maximum of 10mL per bottle. Most samples have a collection < 3mL. |
| Periodicity of BC | No significant difference in yield is expected between multiple cultures obtained simultaneously or those obtained at intervals. |
| Indications for BC | Most patients with septic shock, sepsis or new onset fever should have a BC. Only 10% of BC are positive and a medical prescription is necessary to decide who need BC. |
| Blood culture sampling rate | For detection of nosocomial infection, no threshold is defined but a minimum of 87 BC sets per 1,000 patients-days has been proposed. |
| BC contaminants | Skin organisms can be inoculated into BC if skin disinfection or hand hygiene are not adequately performed. A BCC rate < 3% is expected. |
| **Data before the implementation of the UBC protocol** | |
| Periodicity of BC | BC should be collected by nurses without medical order when fever occurs (above 38.5°c). No medical order is necessary. |
| Blood culture sampling rate | More than 500 BC sets per 1,000 patients-days are collected. |
| Rate of positivity | Less than 5% of BC are positives |
| Local data on BSI | Ten to 15% of patients with a BC have a BSI |
| Local data on BC contaminant | The absolute rate of BCC is around 2% in our ICU |
| Ratio of BCC/BSI | Half of patients with a positive BC don't have a BSI but a BCC |
| **Blood culture sampling procedure** | |
| Unique blood culture bundle | The unique blood culture bundle (table S2) is the new procedure for BC collection:   - use a unique venipuncture for BC - monitor the volume of blood collected - additional BC collection are unnecessary in most cases in particular during subsequent 48 hours. |
| Suspicion of CLABSI | For suspicion of CLABSI, a set of 2 bottles of BC has to be performed simultaneously on the central line and by direct venipuncture to collect a total of 4 bottles. |
| BC: blood culture; CLABSI: central line-associated BSI | |

| **Table S2: Comparison between Multi- sampling and Unique blood culture protocols** | | |
| --- | --- | --- |
| **Procedure** | **Multi-sampling** | **Unique blood culture** |
| **Indication** | BC should be collected by nurses when fever occurs (above 38.5°c). No medical order is necessary. | A medical prescription is mandatory to collect BC. |
| **Infection prevention** | Use alcohol-based hand sanitizer before BC collection.  Use non-sterile gloves.  Use 0.5% chlorhexidine gluconate alcohol for skin disinfection.  Draw blood cultures first after venipuncture. | |
| **Venipuncture** | A venipuncture is performed to collect each BC set (1 aerobic + 1 anaerobic bottle). | A single sample of a large volume of blood (40 mL) is collected through a unique venipuncture and equally distributed into 2 aerobic bottles and 2 anaerobic bottles. |
| **Volume of blood collected** | No optimal volume of blood to collect has been defined. | When collecting a BC, a direct monitoring of the volume of blood collected is recommended. Use the filling mark indicated on the bottle of BC when available or draw one before starting BC collection. Each bottle should be filled with 8 – 10mL of blood. |
| **Interval of sampling and repeated sampling** | Additional BC are drawn without medical order when fever occurs (above 38.5°c) with a maximum of 3 BC sets/day | After a first BC (4 bottles), an additional BC set is strongly discouraged in particular during the next 48 hours. BC must be performed only on medical order for a suspicion of a new BSI. |
| BC: blood cultures; BSI: bloodstream infection | | |

| **Table S3: Knowledge survey about blood cultures, bloodstream infections and contaminants (multiple choice questions)** | |
| --- | --- |
| **Blood culture contaminants** | |
| What is a false positive result? | - A positive result with no bacteremia - A result of Gram-positive bacteria for an enterobacteriaceae - A skin organism inoculated into the BC - A discordance between aerobic and anaerobic bottles - A positive BC without identification of a source of infection |
| What are the potential consequences of BCC? | - A doubt about the diagnosis of BSI - Unnecessary antibiotic prescription - Unnecessary removal of a CVC - Additional cost during hospitalization - Addition length of stay |
| Generalities on BCC | - Each venipuncture is associated with an additional risk of BCC - Antibiotic treatment prevents the risk of BCC - BCC represents more than 1% of BC - More than 10% of positive BC are BCC - BCC are easily distinguished from BSI by direct gram stain |
| **Volume** | |
| The volume collected | - A few drops of blood in each bottle of BC is recommended - The yield of BCC increase with the volume collected - Collecting at least 4 bottles of BC is recommended - Each bottle can be filled with 10 mL of blood - Five seconds are usually sufficient to fill a bottle of BC |
| The volume collected and the optimal diagnostic of BSI | - The yield of BSI increase with the volume collected - Ten milliliters in two bottles equals 10mL in one bottle of BC for the diagnosis of BSI - Forty milliliters of blood collected may diagnose more than 90% of BSI - If a low volume is drawn, a new sample is recommended - Patient with septic shock need less blood collected |
| **Trigger for BC** | |
| Trigger for BC | - Fever is necessary to suspect a BSI - Sepsis is frequently associated with a BSI - Chills are frequently associated with a BSI - A BSI is frequent during community-acquired pneumonia - A BSI is frequent during endocarditis |
| **Pathophysiology of bloodstream infections** | |
| General | - BSI have a low bacterial concentration - During a BSI, bacteremia occurs when chills occur - Most BSI are intermittent - Skin organisms can sometimes be responsible for BSI - Some sources of infections are rarely associated with BSI |
| **Sample procedure** | |
| Hygiene procedure | - Sterile gloves are mandatory - Use of chlorhexidine-alcohol is recommended for skin preparation - Surgical face mask is mandatory during BC collection - The top of blood culture bottles is sterile - Direct venipuncture is safer than a collection through the catheter hub |
| Blood culture collection | - Aerobic BC bottle should be collected before the anaerobic - BC should be collected before biochemical samples - Separate BC sets collections are preferred to diagnose a BSI - Four to six BC set are necessary to best diagnose a BSI - BC collection on CVC limit the risk of BCC |
| BC: blood cultures; BCC : BC contaminants; BSI: bloodstream infection; CVC : Central venous catheter | |

**
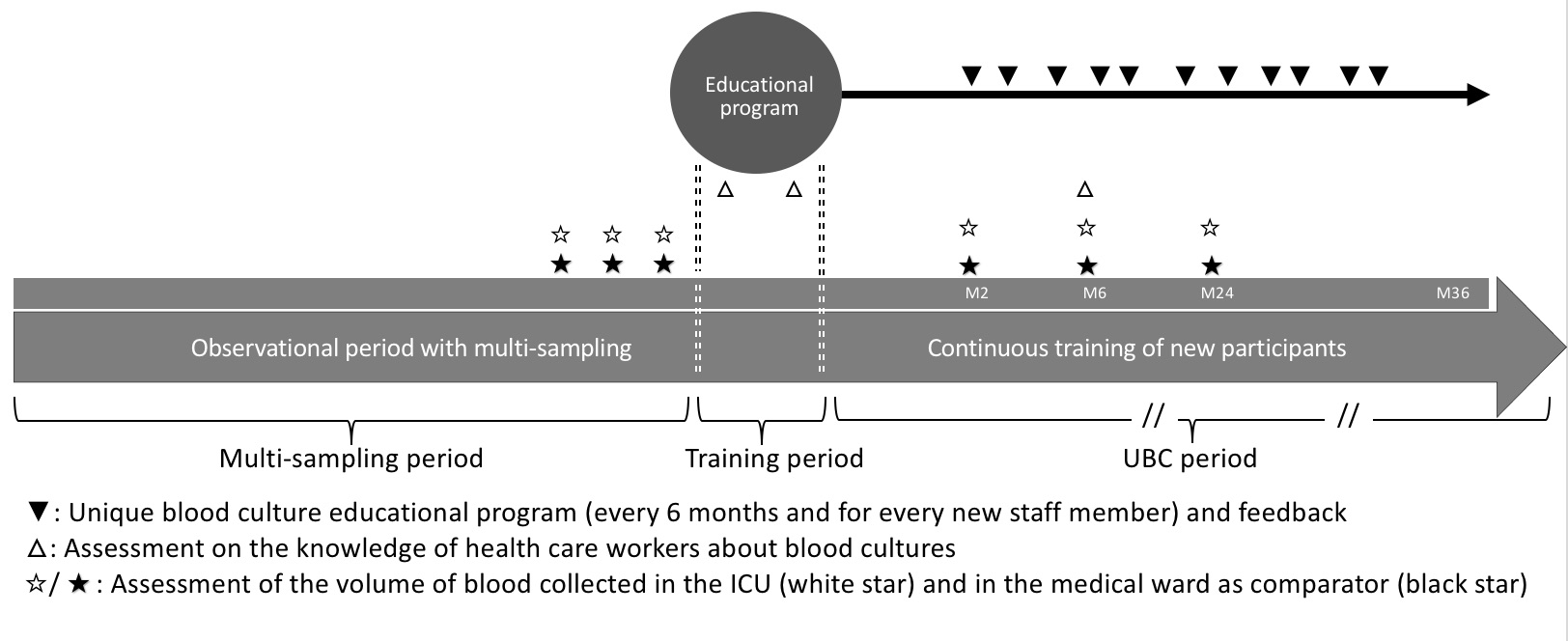
 Figure S1: Schema of our multifaceted educational program for a unique blood culture strategy.**
