# Supplementary material for: Impact of a strategy based on unique blood culture sampling on contamination rate and detection of bloodstream infections in critically ill patients
Source: Ann Intensive Care. 2023 Mar 3;13:13. doi: 10.1186/s13613-023-01107-y (PMC9984630; doi:10.1186/s13613-023-01107-y)
Supplement: Supplementary file 2 — Additional file 2: Figure S2. Evolution of BSI and BC contamination rate during the MS and UBC periods. Figure S3. Proportion of pathogen and contaminant among positive BC. Table S4. Site of acquisition, source of infection and pathogen identifications of BSI. Figure S4. Comparative changes in the mean volume of blood collected per bottle during the MS and UBC periods in the ICU and in the emergency department (ED). Figure S5. Proportion of bacteremia identified according to different volume of blood collected. Figure S6. Mean rate of correct answer for each question of the survey about bloodculture. [file 13613_2023_1107_MOESM2_ESM.docx]

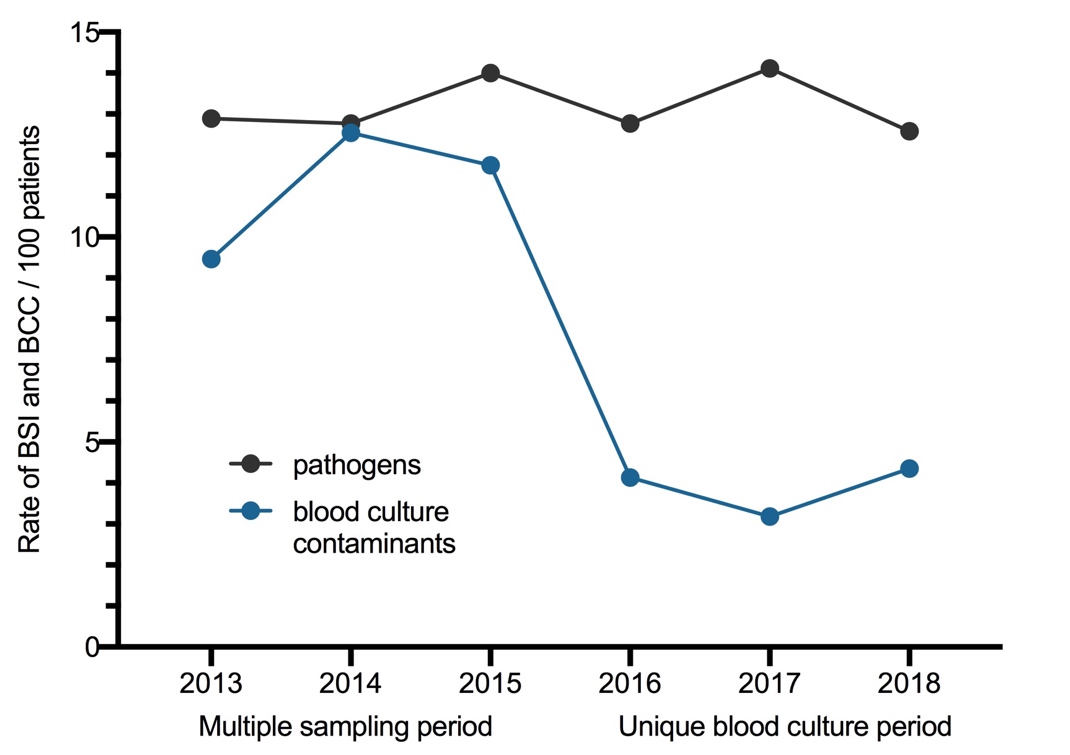


**Figure S2: Evolution of BSI and BC contamination rate during the MS and UBC periods.**

The rate of BSI and BCC are represented for 100 patients with at least one BC collected.

BSI: Bloodstream infections; BCC: Blood culture contaminant.


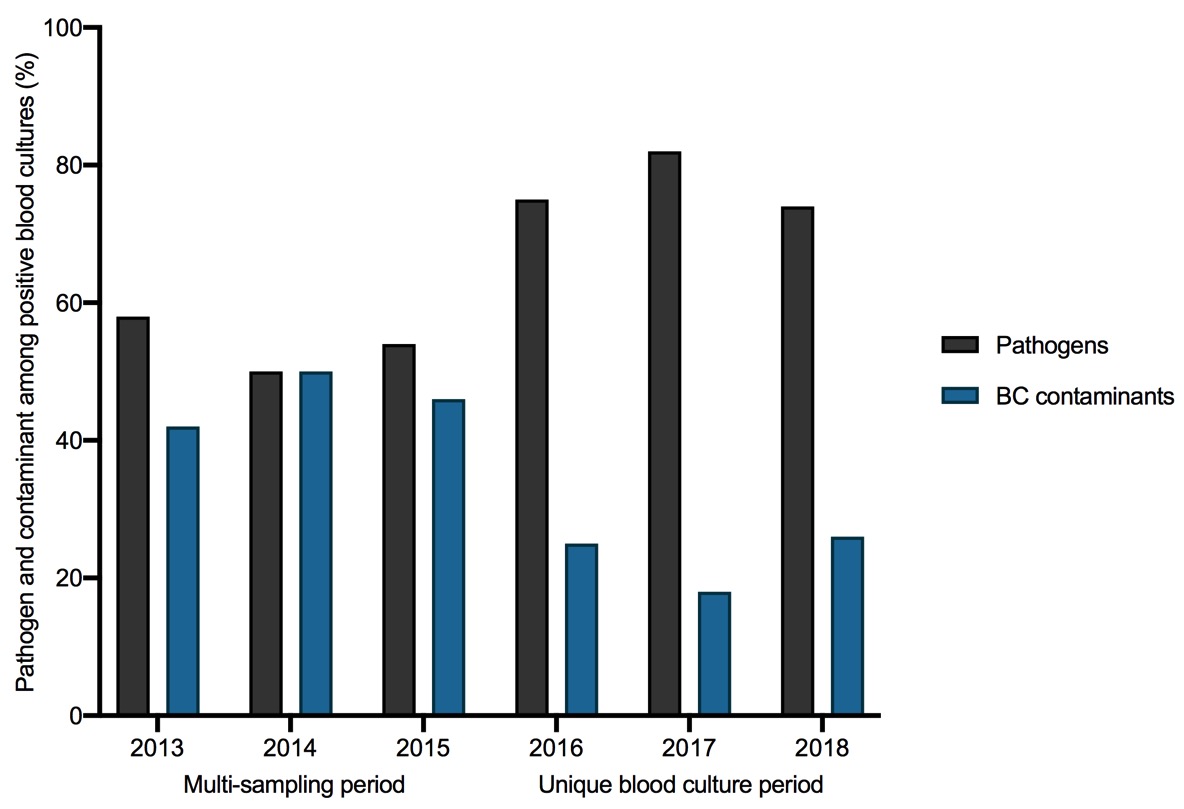


**Figure S3: Proportion of pathogen and contaminant among positive BC.**

| **Table S4: Site of acquisition, source of infection and pathogen identifications of BSI** | | | | |
| --- | --- | --- | --- | --- |
| **Characteristics of BSI** | **Overall** | **MS period** | **UBC period** | **p-value** |
| Site of acquisition |  |  |  | 0.3 |
| Community-acquired | 285 (48.1%) | 171 (49.1%) | 114 (46.7%) |  |
| Hospital-acquired | 122 (20.6%) | 64 (18.3%) | 58 (23.8%) |  |
| ICU acquired | 185 (31.3%) | 113 (32.5%) | 72 (29.5%) |  |
| Source of infection |  |  |  | 0.03 |
| Abdomen | 120 (20.3%) | 66 (19%) | 54 (22.1%) |  |
| Lung | 100 (16.9%) | 62 (17.8%) | 38 (15.6%) |  |
| Primary | 96 (16.2%) | 64 (18.4%) | 32 (13.1%) |  |
| Central line-associated BSI | 92 (15.5%) | 47 (13.5%) | 45 (18.4%) |  |
| Urinary tract | 84 (14.2%) | 57 (16.4%) | 27 (11.1%) |  |
| Endocarditis | 42 (7.1%) | 28 (8%) | 14 (5.7%) |  |
| Cutaneous | 35 (5.9%) | 14 (4%) | 21 (8.6%) |  |
| Oral and upper airway | 21 (3.5%) | 10 (2.9%) | 11 (4.5%) |  |
| Other | 2 (0.3%) | 0 | 2 (0.8%) |  |
| Pathogens |  |  |  | 1 |
| Enterobacteriaceae | 177 (29.8%) | 104 (29.9%) | 73 (29.9%) |  |
| *Staphylococcus aureus* | 114 (19.2%) | 66 (19%) | 48 (19.7%) |  |
| *Streptococcus spp.* | 75 (12.6%) | 43 (12.4%) | 32 (13.1%) |  |
| Polymicrobial | 50 (8.4%) | 27 (7.8%) | 23 (9.4%) |  |
| Anaerobes | 40 (6.7%) | 24 (6.9%) | 16 (6.6%) |  |
| *Enterococcus spp.* | 30 (5.1%) | 20 (5.7%) | 10 (4.1%) |  |
| *Pseudomonas aeruginosa* | 19 (3.2%) | 12 (3.4%) | 7 (2.9%) |  |
| Yeast | 21 (3.5%) | 11 (3.2%) | 10 (4.1%) |  |
| Coagulase negative staphylococcus* | 30 (5.1%) | 16 (4.6%) | 13 (5.3%) |  |
| Other† | 37 (6.2%) | 25 (7.2%) | 12 (4.9%) |  |
| *only CoNS considered as pathogens (with definitive antimicrobial therapy  † Include *Acinetobacter calcoaceticus-baumannii complex (n=4)*, *Campylobacter spp. (n=5), Haemophilus influenzae (n=6), Listeria monocytogenes (n=3), Gemella spp (n=5) (other species with n < 3 are not shown)* | | | | |

**
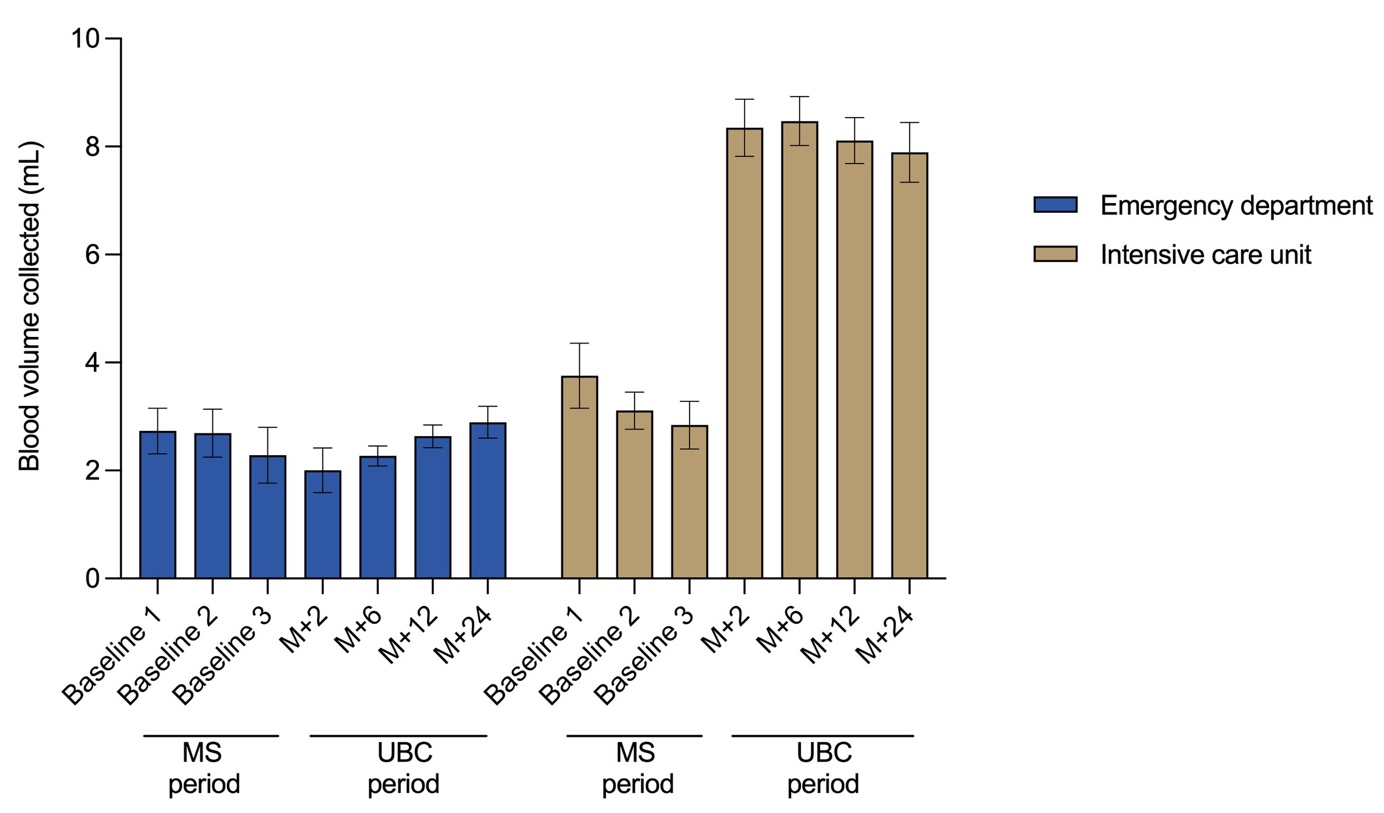
**

**Figure S4: Comparative changes in the mean volume of blood collected per bottle during the MS and UBC periods in the ICU and in the emergency department (ED).**

The I bars represent 95% confidence intervals. MS: multi-sampling; UBC: Unique blood culture.

**
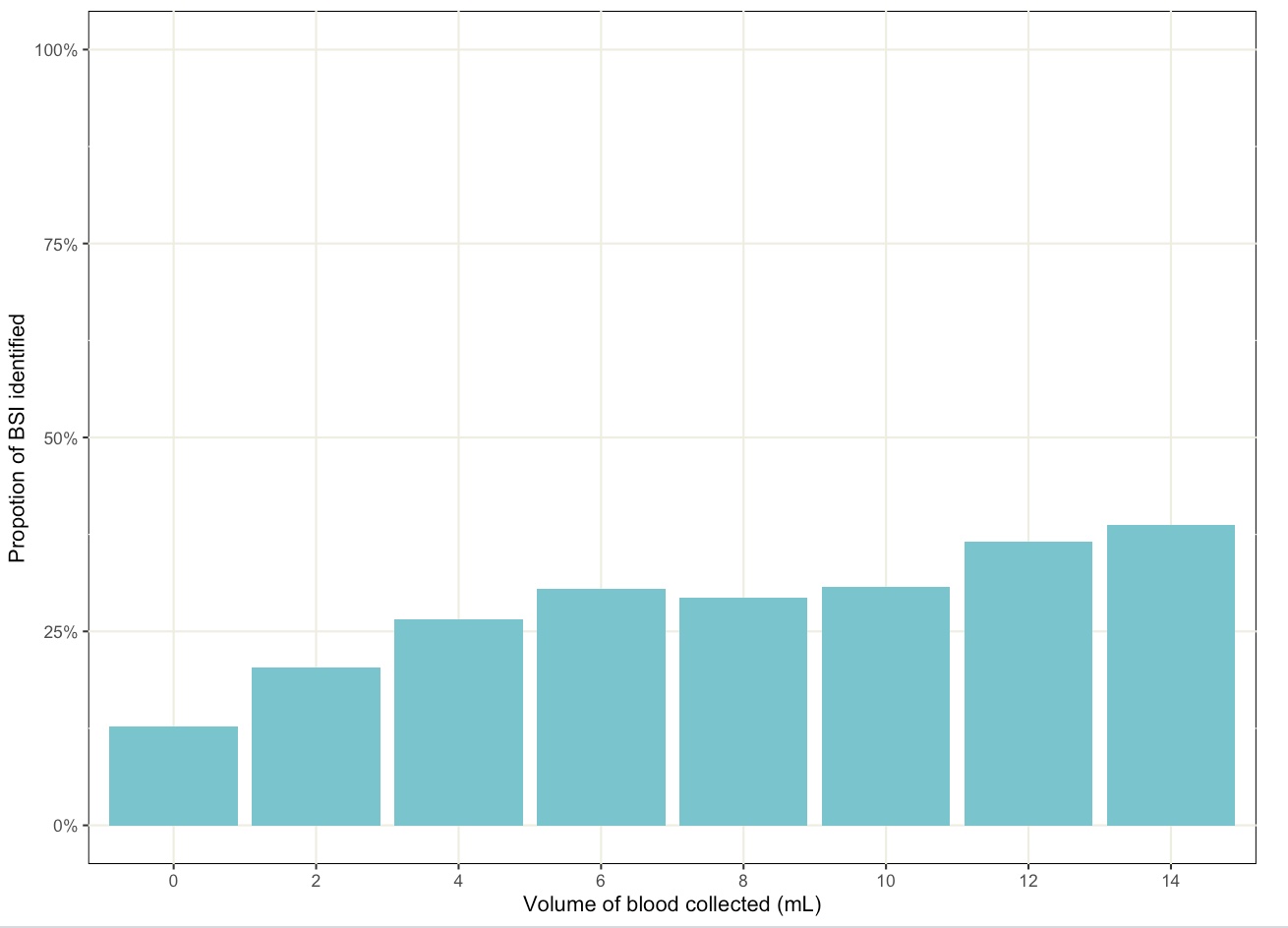
**

**Figure S5: Proportion of bacteremia identified according to different volume of blood collected**


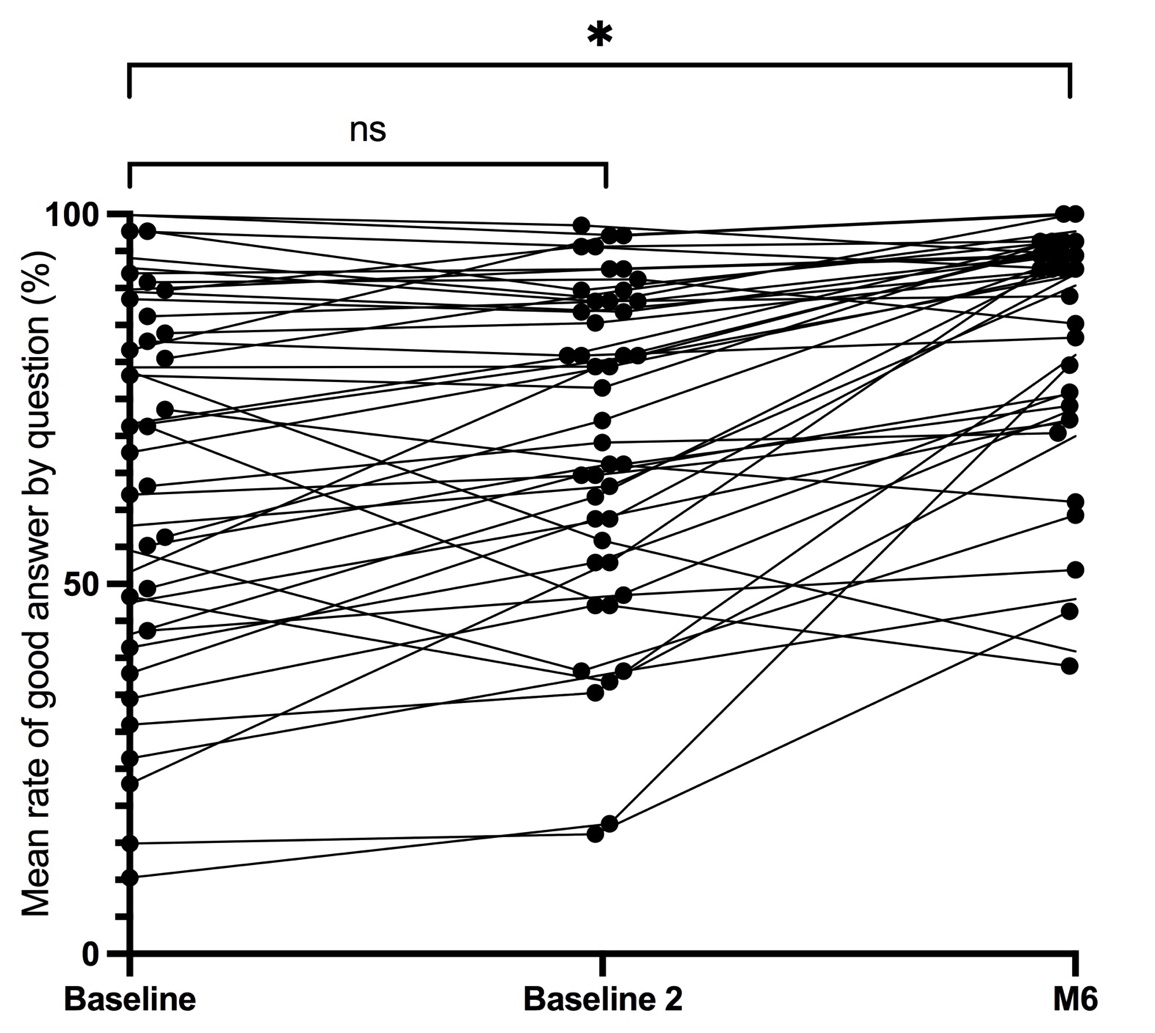


**Figure S6: Mean rate of correct answer for each question of the survey about bloodculture**

No significant difference was observed in the rate of correct answers between the two baseline evaluations (66% and 70% for baseline 1 and 2, respectively; p = 0.6). The mean rate of correct answers increased after the educational program to 83% at the 6-month evaluation (p < 0.001).
